# A199 UTILIZATION OF MUCUS-DERIVED O-GLYCAN SUGARS BY THE PATHOGEN *C. RODENTIUM* FACILITATE ITS COLONIZATION OF THE MURINE GUT

**DOI:** 10.1093/jcag/gwab049.198

**Published:** 2022-02-21

**Authors:** M Mslati, H Yu, C Ma, B Vallance

**Affiliations:** 1 Department of Medicine, BC Children Research Institute, Vancouver, BC, Canada; 2 Paediatrics, Research Institute, BC Children’s Hospital, Vancouver, BC, Canada; 3 BC Children’s Hospital, Vancouver, BC, Canada

## Abstract

**Background:**

The ability of enteric pathogens to colonize and expand within the mammalian gastrointestinal (GI) tract is determined by several factors, including the ability to find and acquire nutrients. The thick mucus layer that lines the inner surface of the large intestine is rich in sugars that can serve as nutrient sources for several members of the microbiota. Whether these sugars can also be used by invading bacterial pathogens to colonize the GI tract is still unclear, in particular for the family of attaching and effacing (A/E) bacterial pathogens, including the human diarrheal pathogens EHEC and EPEC.

**Aims:**

To investigate the ability of the murine A/E pathogen *Citrobacter rodentium* to use mucin-derived sugars as a nutrient source, and the importance of these sugars in the virulence of *C. rodentium* during in-vivo infection.

**Methods:**

To identify which sugar(s) are required for *C. rodentium* to colonize and grow in the murine GI tract, we generated mutants lacking single or multiple genes involved in the uptake and catabolism of mucin-derived O-glycan sugars. This was followed by in-vitro growth assays in minimal media supplemented with mucin sugars to investigate the growth properties of *C. rodentium* and the generated mutants on mucin sugars.

**Results:**

We determined that *C. rodentium* was able to use three mucin O-glycan sugars: sialic acid, galactose, and N-acetylglucosamine (GlcNAc) as both carbon and nitrogen sources for in-vitro growth. *C. rodentium* exhibited the maximal growth rate and density on GlcNAc, followed by sialic acid, and finally galactose. A mutant *C. rodentium* strain carrying a deletion in the nagA gene was unable to grow on both GlcNAc and sialic acid, confirming that the breakdown pathways for these two sugars merge and are processed by shared suite of enzymes. As for galactose, combined deletions in the genes mglB and galP were required to abolish growth on this sugar. Notably, a mutant strain carrying simultaneous deletions in nagA, mglB, and galP was unable to grow on all three mucin sugars, as well as on purified mucin.

**Conclusions:**

Our results demonstrate that intestinal mucin sugars serve as potential nutrient sources for *C. rodentium* and that *C. rodentium* can catabolize three of these sugars. Future work will examine whether these sugar pathways contribute to *C. rodentium* colonization of the murine GI tract.

**Funding Agencies:**

CCC, CIHRCH.I.L.D. Fdn

